# Gene expression in reproductive organs of tsetse females – initial data in an approach to reduce fecundity

**DOI:** 10.1186/s12866-018-1294-5

**Published:** 2018-11-23

**Authors:** Emanuel Procházka, Veronika Michalková, Ivana Daubnerová, Ladislav Roller, Peter Klepsatel, Dušan Žitňan, George Tsiamis, Peter Takáč

**Affiliations:** 1 Institute of Zoology, Slovak Academy of Sciences, Dúbravská cesta 9, 845 06 Bratislava, Slovakia; 2 Department of Environmental and Natural Resources Management, University of Patras, 2 Georgiou Seferi St, Agrinio, Greece; 3 Scientica, Ltd., Hybešova 33, 831 06 Bratislava, Slovakia

**Keywords:** Tsetse, Transcriptome, Fold change, Ovaries, Uteri

## Abstract

**Background:**

Tsetse flies are vectors of African trypanosomes, and their vectorial capacity results in a major public health emergency and vast economic losses in sub-Saharan Africa. Given the limited ability of trypanosome prevention and eradication, tsetse vectors remain major targets of control efforts. Larvae of all three instars are developed in mothers’ uteri, nourished through milk, and ‘larviposited’ shortly before pupation. The past few years have witnessed the emergence of approaches based on knockdown of genes involved in milk production, resulting in a significant reduction of fecundity.

**Results:**

In order to identify further genes applicable in the control of tsetse flies, we determined the expression of protein-coding genes in ovaries and uteri from both virgin and heavily pregnant *Glossina morsitans morsitans* females. Comparison of expression profiles allowed us to identify candidate genes with increased expression in pregnant individuals. Lists with the highest increases include genes involved in oocyte and embryonic development, or nourishment. Maximum ovarian fold change does not exceed 700, while the highest uterine fold change reaches to more than 4000. Relatively high fold changes of two neuropeptide receptors (for corazonin and myosuppressin) propose the corresponding genes alternative targets.

**Conclusions:**

Given the higher fold changes in the uterus, targeting gene expression in this tissue may result in a more evident reduction of fecundity. However, ovaries should not be neglected, as manifested by several genes with top fold changes involved in early developmental stages. Apart from focusing on the highest fold changes, neuropeptide receptors with moderate increases in expression should be also verified as targets, given their roles in mediating the tissue control. However, this data needs to be considered initial, and the potential of these genes in affecting female fecundity needs to be verified experimentally.

**Electronic supplementary material:**

The online version of this article (10.1186/s12866-018-1294-5) contains supplementary material, which is available to authorized users.

## Background

Human (HAT) and animal (AAT) African trypanosomiases are serious diseases caused by unicellular protozoan parasites belonging to the genus *Trypanosoma*. Some livestock breeds and wild animals are trypanotolerant, but in humans, untreated HAT eventually leads to death [1, 2]. In addition to this, treatment of advanced HAT is complicated, relying on either toxic arsenic-based melarsoprol, or a combination of nifurtimox and eflornithine, which requires complex administration [3, 4]. Apart from all of these difficulties, trypanosomiases negatively interfere with people’s socio-economic status. AAT impacts meat and milk production, and regarding the agriculture, it also reduces availability of animals providing draft power and fertilizing the soil [5]. The number of individuals infected with HAT dropped rapidly in past years. While there were historically more than 300,000 new HAT cases annually in sub-Saharan Africa, extensive control efforts have helped to reduce this number to less than 3000 cases in 2015. However, the estimated population at risk reaches to 65 million people [6, 7]. History has taught us that the neglect of appropriate control leads to resurgence of the disease, up to an epidemic scale. Therefore, in order to prevent another rise of HAT in the future, effective control must be continuously exerted [8, 9].

Trypanosomes use tsetse flies from *Glossina* genus (Diptera: Glossinidae) to accomplish their transmission. Tsetse flies are obligatory blood feeders, and they acquire the pathogens while feeding on infected animals (livestock, wildlife) or humans. Once the fly becomes infective, it remains so for the rest of its life, delivering trypanosomes into another host during the taking of a blood meal [4, 10, 11]. Tsetse flies are adenotrophic viviparous organisms, which is a rare trait in Diptera. The embryo and then the larva is developed in the mother’s uterus and nourished through secretions from milk glands connected to the uterine tissue. The 3rd instar larva is deposited shortly (less than 2 h) before pupation, and metamorphosis to imago is completed in 30 days from parturition. As only a single offspring is produced during each gonotrophic cycle, each female gives birth to 8–10 progeny during her life (reviewed in [12]).

Tsetse flies, due to their being exclusive vectors of trypanosomes [13], are attractive targets for trypanosomiasis control. This is even strengthened by the emergence of resistance against both veterinary and human drugs [4, 14], and the unavailability of vaccines. Insecticides are currently used only in low amounts, and their use is usually followed by the environmentally friendly Sterile insect technique (SIT) [15, 16]. However, it is likely that in the future, further improvement over the powerful, yet still random-based SIT, will employ the direct knockdown of selected genes. There are several papers demonstrating reduced fecundity following knockdown of genes involved in lipid [17, 18] or protein [19–22] contribution to milk. Decreased fecundity, partly caused by inadequate milk production, is typical also of females lacking superoxide dismutase activity [23]. With the advance of next-generation sequencing techniques, the genetic manipulation has been facilitated by the availability of the complete genome sequence from tsetse fly *Glossina morsitans morsitans* [24].

In this work, we determined the expression of protein-coding genes in ovaries and uteri from both virgin and heavily pregnant *G. m. morsitans* flies. As these tissues are directly responsible for reproduction, they are suitable objects of further search for candidate genes applicable for the reduction of fecundity. Comparison of expression profiles between pregnant and virgin females allowed us to identify several genes, which can be further tested as targets for RNA interference. Besides this, very little is known about genes expressed in ovaries and uteri of tsetse females. Hens et al. [25] identified a yolk protein gene in *G. m. morsitans*, whose product apparently serves as proteinaceous nutrient for the embryo. The gene is expressed exclusively by ovarian follicle cells [25]. Apart from this, several other papers have described transcription of various genes in the reproductive tract [26–28], but the more detailed localization of their expression was not identified. Our results, therefore, also shed more light on gene expression in these tissues.

## Methods

### Flies

*G. m. morsitans* flies were reared at the Tsetse Research and Mass Rearing Facility, Institute of Zoology, Slovak Academy of Sciences, Bratislava, Slovakia. Flies were maintained at 24 ± 1 °C with 74–75% relative humidity, fed on blood meal provided through an artificial feeding system at 48 h intervals, and starved for 2 days before dissection. 5-day old females were used as virgins, and primiparas containing 3rd instar larvae (not more than a couple of hours before parturition) were used as pregnant. These heavily pregnant females were identified visually according to visible or protruding black larval spiracles.

### RNA isolation, transcriptome sequencing and analysis

Every condition was analyzed in a single replicate (i.e. a total of four samples were sequenced). Ovaries for each ovarian sample were dissected from 20 individuals. Uteri for each uterine sample were (due to lower RNA content) collected from 50 individuals. Organs were dissected in solution containing: 140 mM sodium chloride, 5 mM potassium chloride, 1 mM magnesium chloride, 5 mM calcium chloride, 4 mM sodium hydrogencarbonate, and 5 mM 4-(2-hydroxyethyl)-1-piperazineethanesulfonic acid (HEPES); pH was adjusted to 7.2 using sodium hydroxide or hydrochloric acid. Dissected tissues were temporarily stored in RNAlater (Sigma-Aldrich) at − 20 °C. Total RNA for each sample was isolated from pooled tissues using RNeasy Protect Mini Kit (QIAGEN), including the on-column deoxyribonuclease treatment to remove the residual DNA. RNA samples were further processed by a Microsynth sequencing facility (Balgach, Switzerland). Libraries were constructed using TruSeq RNA Library Prep Kit v2 (includes poly(A) enrichment), and sequenced on Illumina NextSeq 500 platform (Illumina, San Diego, CA, USA). Numbers of generated past filter reads and bases (passing initial Illumina read processing) and corresponding quality scores are presented in Table 1. Sequencing data are available in Sequence Read Archive (SRP137614).

**Table 1:** Sequencing and quality score statistics

sample	past filter reads	past filter bases	mean read length	mean Q20%	mean Q30%	mean Q
ovaries - virgin	26,010,484	1,937,796,430	75	97,49	96,18	35
ovaries - pregnant	25,785,178	1,920,965,446	75	97,43	96,09	35
uteri - virgin	20,844,784	1,552,325,258	74	97,37	96,02	35
uteri - pregnant	26,375,428	1,961,563,862	74	97,33	95,95	35

Past filter reads were applied to tag counting analysis (done by Microsynth). Reads were mapped to a *G. m. morsitans* reference genome (as available from www.vectorbase.org on 31st August 2017) using STAR (v 2.5.1b). This produced a pool of uniquely mapped reads (mapped to a single site). Uniquely mapped reads were further assigned to annotated genes and counted using HTSeq (v 0.6.0).

Gene counts derived from corresponding tissues (e.g. ovaries of virgin and pregnant flies) were used to calculate *P*-values and fold changes. To assess the differences in gene expression, we used Fisher’s exact tests. *P*-values were adjusted for multiple testing by using the Bonferroni correction. Changes in gene expression are represented as fold changes. Fold change (FC) for each gene is determined as the ratio of normalized gene count from pregnant female vs. normalized gene count from virgin. FC > 1 then represents higher expression in pregnant flies, while FC < 1 means higher expression in virgins. Two genes, *TBB1* (GMOY000148) and *GAPDH* (GMOY000473), were used for normalization, albeit in separate calculations (for more information, see Results).

*G. m. morsitans* gene IDs, gene names, and gene descriptions, and homologues from *Drosophila melanogaster* are listed in Additional file 1: Table S1. Gene product names in Tables 5 and 6 correspond to gene descriptions in Additional file 1: Table S1, or have been retrieved from flybase.org according to their homology to *D. melanogaster* (Additional file 1: Table S1), except for three genes (GMOY008375, GMOY004923, and GMOY009721) whose products have been predicted manually using BLAST tools available at blast.ncbi.nlm.nih.gov/blast.cgi. All gene counts, *P*-values, and calculated FCs are listed in Additional file 2: Table S2 and Additional file 3: Table S3. Lists with FCs arranged in descending order contain only genes with *P* < 0.05. Expression data on neuropeptide receptors are summarized in Additional file 4: Table S4.

## Results

### General features of sequencing and tag counting analysis

Illumina sequencing generated more than 20 million past filter reads (corresponding to more than 1.5 billion past filter bases) for each sample. More than 95% of past filter bases in each sample reached a base call accuracy of 99.9% (Q30%), and each mean Q score was equal to 35 (Table 1). The past filter reads were then mapped to the *G. m. morsitans* genome and trimmed to remove mainly multi-mapping reads, reducing the total number of reads to 84.5–93.1%. Resulting reads that uniquely mapped to the genome were further trimmed to remove reads that did not map to annotated genes, or were mapped to them ambiguously. Final reads uniquely mapped to annotated genes constituted 61.6–76.7% of the initial past filter reads (Table 2).

**Table 2:** Mapping statistics

sample	past filter reads	reads uniquely mapped to genome	reads uniquely mapped to annotated genes
ovaries - virgin	26,010,484	23,943,403 (92.1%)	19,398,018 (74.6%)
ovaries - pregnant	25,785,178	24,002,608 (93.1%)	19,768,150 (76.7%)
uteri - virgin	20,844,784	18,542,508 (89.0%)	14,049,211 (67.4%)
uteri - pregnant	26,375,428	22,298,336 (84.5%)	16,257,774 (61.6%)

% = per total number of past filter reads generated for the given sample

### Reference genes

*TBB1* gene coding for β-tubulin is the most widely used reference (housekeeping) gene for normalization of expression data from *Glossina* (e.g. [29]). Another, although less widely employed gene, codes for glyceraldehyde 3-phosphate dehydrogenase I (*GAPDH*) (e.g. [30]). We used both of these genes to normalize the counts of mapped reads. However, at least one of them was not expressed uniformly as indicated by highly divergent uterine ratios (see Table 3, in bold). From the obtained data, we were unable to determine which gene is more appropriate, therefore we used both genes for normalization, albeit in separate calculations.

**Table 3:** Read counts and calculated ratio for selected reference genes

sample	*TBB1* read counts	*GAPDH* read counts	count ratio (*TBB1*: *GAPDH*)
ovaries - virgin	59,949	12,866	4.66
ovaries - pregnant	58,658	11,607	5.05
uteri - virgin	78,190	3835	**20.39**
uteri - pregnant	98,139	3157	**31.09**

Highly divergent uterine ratios are highlighted in bold

### General features of fold changes

Normalized gene counts were used for the calculation of FCs. Although FC was formally calculated for every gene, we further considered only genes whose *P*-values were lower than 0.05. This reduced the total number of genes from 12,850 to 4067 (ovaries), and to 4095 (uteri), respectively. Although the numbers are similar, in fact only 1987 genes are shared. In our FC calculations, we did not apply the correction for gene length, since each calculation used only gene counts corresponding to the same gene.

**Fig. 1:**
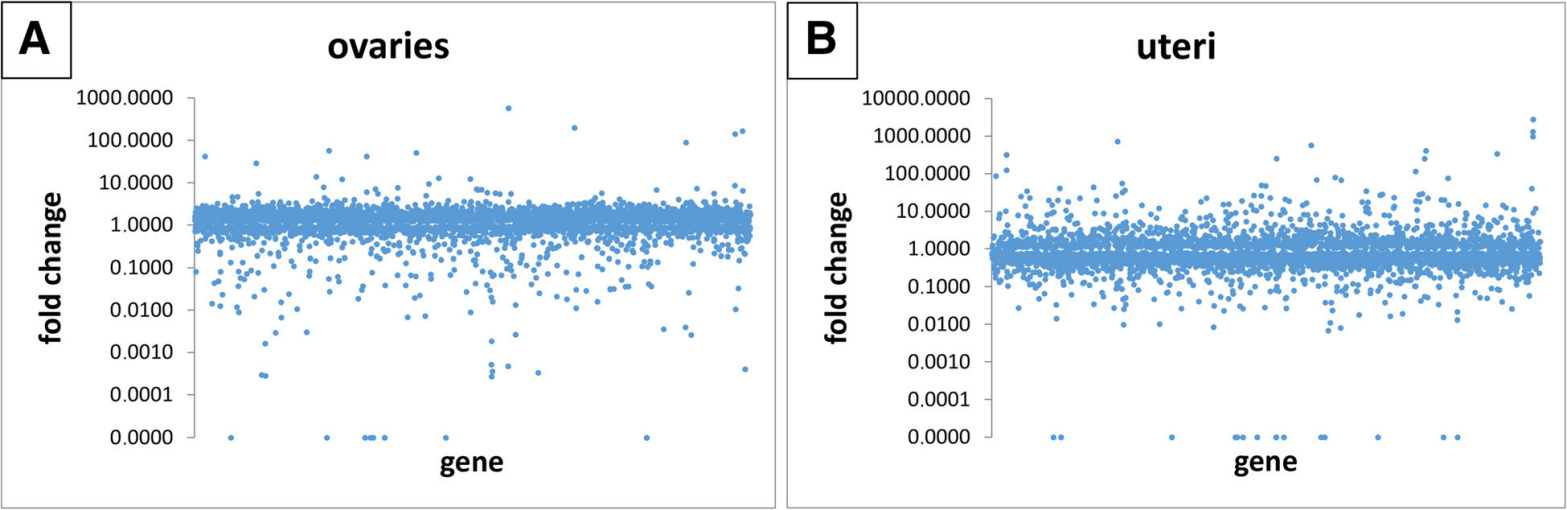
Examples of scatter plots – ovarian (**a**) and uterine (**b**) fold changes**.** Genes with *P* < 0.05 are plotted (ovaries – 4061; uteri – 4072). Only *TBB1*-normalized FCs are depicted

Distribution of FCs is represented in Table 4. The highest increases are found in uterine expressions. For example, there are four genes with FC >  100 found in ovaries, but 12 or 17 in uteri. The same imbalance is true also for FCs > 10. But generally, genes with a high increase constitute only a very small portion of the total number of genes. Few genes (referred to as N/A in Table 4) do not have an assigned FC, as the calculation cannot be applied to data with initial zero values (due to the impossibility of division by zero). Examples of scatter plots are shown on Fig. 1.

**Table 4:** Distribution of fold changes – number of genes per fold change interval

FC → sample ↓	> 100	100..10	10..2	< 2	N/A
ovaries (*TBB1* norm.)	4 (0.10%)	10 (0.25%)	586 (14.4%)	3461 (85.1%)	6 (0.15%)
uteri (*TBB1* norm.)	12 (0.29%)	71 (1.73%)	358 (8.74%)	3631 (88.7%)	23 (0.56%)
ovaries (*GAPDH* norm.)	4 (0.10%)	11 (0.27%)	799 (19.6%)	3247 (79.8%)	6 (0.15%)
uteri (*GAPDH* norm.)	17 (0.42%)	107 (2.61%)	795 (19.41%)	3153 (77.0%)	23 (0.56%)

Only genes with *P* < 0.05 were considered. % = per total number of genes with *P* < 0.05 (ovaries – 4067; uteri – 4095); N/A = not calculated due to initial zero count

**Fig. 2:**
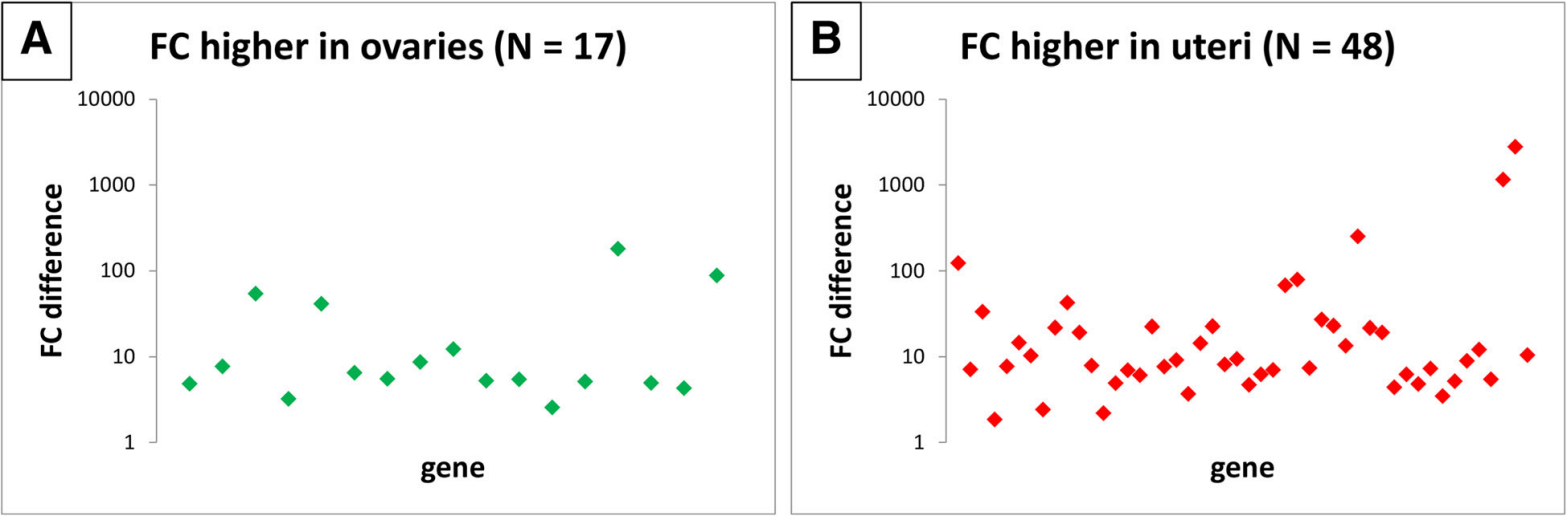
Difference between ovarian and uterine fold changes determined for the same gene – *TBB1* normalization. Fold change (FC) difference is calculated between ovarian FC and uterine FC, and is expressed as an absolute value. If ovarian FC is higher than uterine FC, the gene is plotted in (**a**); otherwise in (**b**). Genes with both ovarian and uterine *P*-values < 0.05, and with FC > 5 (either ovarian or uterine) are depicted. N = number of genes; total number of analysed genes = 65

**Fig. 3:**
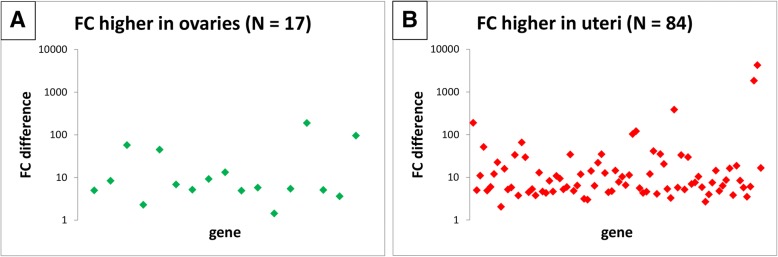
Difference between ovarian and uterine fold changes determined for the same gene – *GAPDH* normalization. Fold change (FC) difference is calculated between ovarian FC and uterine FC, and is expressed as an absolute value. If ovarian FC is higher than uterine FC, the gene is plotted in (**a**); otherwise in (**b**). Genes with both ovarian and uterine *P*-values < 0.05, and with FC > 5 (either ovarian or uterine) are depicted. N = number of genes; total number of analysed genes = 101

The numerical dominance of the higher uterine FCs over the ovarian ones is illustrated by Figs. 2 and 3. We compared ovarian and uterine FCs determined for the same gene. Such a comparison could have been applied only to genes with both ovarian and uterine *P*-values smaller than 0.05. To exclude the majority of the smallest differences in FCs we used only genes with FC > 5 (either ovarian or uterine). Finally, we calculated the difference between ovarian and uterine FCs corresponding to the same gene. One can easily notice that for the majority of analysed genes the uterine FC is higher than its ovarian counterpart.

### Detailed look at the genes with the highest increase in expression

The following Tables 5 and 6 list genes whose expressions exhibited the highest FCs. Specifically, we were curious as to whether they are known players in the reproductive process (addressed in more detail in Discussion), and as to the differences between ovarian and uterine top genes. Every table contains ten top genes.

**Table 5:** Top 10 ovarian fold changes

gene ID	(predicted) gene product	FC (*TBB1* norm.)	FC (*GAPDH* norm.)
GMOY006879	insulin-like peptide 8 (Ilp8)	577.55	626.41
GMOY008375	hydroxysteroid dehydrogenase-like protein 1 (HSDL1)	199.29	216.15
GMOY012369	milk gland protein 10 (Mgp10)	165.57	179.57
GMOY012016	milk gland protein 8 (Mgp8)	142.06	154.08
GMOY010882	chemosensory protein 3 (CSP3)	88.91	96.44
GMOY002936	cuticular protein 67B	57.23	62.07
GMOY004923	cuticular protein 16.5, isoform A-like	51.10	55.42
GMOY000264	Krüppel protein (Kr)	41.90	45.45
GMOY003840	Obstructor-E (Obst-E)	41.90	45.45
GMOY001345	*unknown*	28.96	31.41

Only genes with *P* < 0.05 were considered. Calculated FCs were arranged in descending order and ten highest are listed. The top position is represented by gene exhibiting highest fold change in expression between ovaries of pregnant and virgin females. The full list can be found in Additional file 2: Table S2

**Table 6:** Top 10 uterine fold changes

gene ID	(predicted) gene product	FC (*TBB1* norm.)	FC (*GAPDH* norm.)
GMOY012377	milk gland protein 4 (Mgp4)	2798.11	4266.24
GMOY012369	milk gland protein 10 (Mgp10)	1328.14	2025.00
GMOY012371	milk gland protein 9 (Mgp9)	983.96	1500.23
GMOY002808	*unknown*	724.46	1104.58
GMOY007129	fatty acid 2-hydroxylase	575.24	877.06
GMOY009745	milk gland protein 1 (Mgp1)	415.09	632.89
GMOY011400	cyp313b1	344.76	525.65
GMOY000398	phantom protein (Phm)	320.28	488.33
GMOY006331	*unknown*	255.42	389.44
GMOY009721	Stall protein (Stl)	254.16	387.51

Only genes with *P* < 0.05 were considered. Calculated FCs were arranged in descending order and ten highest are listed. The top position is represented by gene exhibiting highest fold change in expression between uteri of pregnant and virgin females. The full list can be found in Additional file 3: Table S3

The highest FC in uteri reaches almost 3000 (*TBB1* norm.), and more than 4000 (*GAPDH* norm.), respectively. This is several times higher compared to top genes from ovaries (577.55; 626.41). Generally, the FCs are higher than their ovarian counterparts listed at the same position. The tables share one gene (coding for Mgp10), but otherwise are different.

### Expression of neuropeptide receptors

We further analysed the expression of neuropeptide receptors. Neuropeptides act as neuromodulators in the central nervous system, and as regulatory hormones released into the circulation activate receptors in peripheral tissues. Neuropeptide receptors produced in ovaries and uteri could therefore provide alternative targets for gene knockdown, since silencing of their expression may unbalance the control and function of target tissue. We took a closer look at 33 genes putatively coding for neuropeptide receptors. Out of these, five putative receptor genes exhibited increased expression in pregnant females at *P* < 0.05. Expression of genes coding for corazonin and myosuppressin receptors reached FCs > 20 (Table 7). The full list with 33 genes and corresponding data is presented in Additional file 4: Table S4.

**Table 7:** Neuropeptide receptor genes with fold change > 1

gene ID	predicted gene product	OVARIES		UTERI	
		FC (*TBB1* norm.)	FC (*GAPDH* norm.)	FC (*TBB1* norm.)	FC (*GAPDH* norm.)
GMOY006527	corazonin receptor	–	–	21.19	32.31
GMOY006636	short neuropeptide F receptor	–	–	6.19	9.43
GMOY008798	SIFamide receptor	2.94	3.19	–	–
GMOY009062	CCAP receptor	5.67	6.15	0.70	1.07
GMOY009670	myosuppressin receptor	–	–	29.48	44.95

Genes are arranged by gene ID; “–” means that FC is not applied due to *P* > 0.05

## Discussion

Considerable achievements have been made in an effort toward establishing an environmentally friendly method of vector control employing knockdown of selected genes (see Background). Tables 5 and 6 give us hints at which genes expressed in the female reproductive tract could be further targeted. We observed great expression changes in both ovaries and uteri. Uterine FCs (Table 6) are even higher than their ovarian counterparts listed at the same position (Table 5), which proposes uteri are (in numerical terms) a more suitable target for further knockdown experiments.

Some candidate genes have not been described yet, or lack considerable homology, and are therefore referred to as unknown. On the contrary, several genes can be grouped based on their function, and a lot of them are known to be related to the reproductive process in tsetse or other organisms (see below).

Ovaries used for RNA extraction from both virgin and pregnant flies contained oocytes. However, we suppose that genes expressed in oocytes provide extra targets for gene knockdown, aiming directly at the developing offspring. This could be the case of gene coding for insulin-like peptide 8 (Ilp8), having the highest ovarian FC. In *Drosophila*, Ilp8 is expressed by growing tissues during development, and coordinates their growth status with developmental timing (i.e. synchronizes growth between different organs). Therefore, it is likely that observed expression and FC should be assigned to the developing egg. Nevertheless, loss of Ilp8 in *Drosophila* results in delayed pupation, while developed animals exhibit considerable variation in final size and imperfect bilateral symmetry [31, 32]. Krüppel protein (Kr) is a similar case to Ilp8, as its transcriptional data most likely originate in the developing egg. It is also directly involved in the development in *Drosophila*, serving as a transcription factor, and being expressed during the blastoderm stage of embryogenesis [33]. Finally, Table 5 contains group of cuticular proteins (67B, 16.5, and Obst-E), which are apparently also of embryonic origin. Absence of Obst-E in *Drosophila* results in deficient larval cuticle and misshaped puparium [34].

The second highest ovarian FC corresponds to gene coding for hydroxysteroid dehydrogenase-like protein 1 (HSDL1). In humans, HSDL1 is highly expressed in testes and ovaries. Hydroxysteroid dehydrogenases play an important role in sex differentiation, or the emergence and maintenance of the secondary sexual characters [35]. Its homologue from *Drosophila* has not been characterized yet.

The ovarian top 10 table is almost totally different from its uterine counterpart. However, Milk Gland Proteins (MGPs) are present in both top 10 lists, occupying two places in ovarian Table 5, and four places in uterine Table 6. One can easily notice that uterine FCs for MGPs are much higher than the values obtained from ovaries.

Proteins are, along with lipids, major constituents of tsetse milk [36]. In *G. m. morsitans*, the most abundant protein is Milk Gland Protein 1 (Mgp1), accounting for more than 90% of protein content [37]. Mgp2–10 were identified according to the presence of corresponding mRNAs in milk gland tissue, and further confirmed by proteomic analysis conducted on larval gut contents [22, 38].

Proteins found in milk are synthesized by secretory cells of the milk glands, and stored in dedicated reservoirs before their final release into milk gland lumen. Milk glands are connected to the uterus, allowing the delivery of milk to the larva (reviewed in [12]). Therefore, the extremely increased expression levels of the two MGPs observed in ovaries are unexpected. On the other side, although the tissues were dissected employing the best practice, high MGP expression changes in uteri may be derived from residual milk glands. However, Mgp1, known as the most abundant protein in milk, is (in Table 6) far behind the other three MGPs, which suggests that the obtained data is genuine to uterus.

Fecundity is lowered by 50% in flies lacking Mgp1 [19]. Concerning Mgp2–10, knockdown of a single MGP gene does not influence the fecundity. On the other hand, the lack of two MGPs reduced the fecundity by 10–15%, while simultaneous knockdown of four MGP genes reduced it by nearly 70% [22]. Neither Mgp4 (topping the Table 6) nor Mgp10 (present in both Tables 5 and 6) were involved in those experiments.

Table 6, listing uterine FCs, contains genes whose homologues from *Drosophila* code for phantom protein and Stall protein, respectively. Absence of these proteins in *Drosophila* results in defects in oogenesis [39, 40]. Presence of the corresponding genes in the uterine top 10 list is rather surprising, but indicates that these products are necessary also in later stages of embryogenesis in *Glossina*.

Finally, we observed that genes coding for corazonin and myosuppressin receptors exhibited rather higher uterine FCs (> 20). Corazonin is considered a stress-induced hormone that participates in numerous processes in insects (reviewed in [41]). For example, knockdown of its receptor improves the resistance to starvation in *Drosophila* [42]. A significant link of corazonin to reproduction has been revealed recently by Gospocic et al. [43]. Lack of this peptide led to increased expression of vitellogenins (proteins important for egg development), and to higher egg-laying rate in *Drosophila*. Based on this, knockdown of the corazonin receptor may cause an increased rate of larviposition in *Glossina*. Although this is in opposition to an effort to reduce the fecundity of the fly, the larvae may not be properly developed due to inadequate time spent in the mother’s uterus. Absence of corazonin signalling may therefore lead to production of unviable progeny.

*Drosophila* myosuppressin (also known as dromyosuppressin) is widely recognized as an inhibitor of muscle activity. Although its physiological role remains unknown, it substantially decreases the heart rate [44, 45], and crop contractions in the fruit fly [46]. Dromyosuppressin was also found to slow the crop movements in the blow fly, *Phormia regina* [47]. Heifetz et al. [48] observed changes in immunoreactivity to myosuppressin in the innervation of reproductive tract in *Drosophila* females during and after mating. This suggests that this neuropeptide may play some role in fertilization and oviposition.

It should be noted that all peptide receptors expressed at substantial level in female reproductive system deserve our attention as potential targets for gene silencing experiments. The activity of reproductive organs can be affected by neuropeptides released at specific time depending on mating status and / or stage of pregnancy. Therefore the expression of corresponding receptors in target organ does not have to significantly change after mating. However, in the absence of additional data, the two mentioned receptors are the most suitable targets.

Apart from identification of suitable genes, a convenient way of their turning off must be determined. In laboratory experiments, dsRNA is delivered into the adult female by injection. Naturally, this is not sustainable in large-scale mass production. Walshe et al. [49] demonstrated that dsRNA could be easily delivered into the tsetse fly during feeding on blood meals. However, it might fail to silence some genes [49]. As an alternative method, paratransgenesis could be exploited [50]. In this strategy, symbionts naturally occurring in their hosts are genetically manipulated to reduce vector competence [51]. Tsetse flies contain up to three different symbionts. Of these, *Sodalis glossinidius* is the most suitable for genetic modification, as methods for its cultivation and transformation have already been developed. This symbiont is present in milk, which allows the direct interaction with offspring. Although the symbiont is found also in other tsetse organs, it encodes genes which are apparently expressed preferentially in the milk glands and during early development of the larva (reviewed in [52]). Thus, utilizing their promoter elements might allow site-specific silencing of those tsetse genes, which are vital for the mother. Nevertheless, construction of a stable paratransgenic *Glossina / Sodalis* line would require an appropriate expression system, blocking the silencing dsRNA expression during propagation of flies in a mass rearing facility.

## Conclusions

In this work, we obtained initial transcriptomic data on the gene expression in reproductive organs of *G. m. morsitans* females. We compared the expression levels in virgin and pregnant flies and identified genes with elevated FCs in both ovaries and uteri. Exactness and significance of this data is limited by the fact that only a single replicate was employed in each condition, and that the virgins were not age-matched to their pregnant counterparts. Although 5-day old virgins reflect the situation in nature better than older ones, the significance of identified genes needs to be validated. Furthermore, potential of these genes in affecting female fecundity needs to be verified experimentally. Nevertheless, many genes listed in this work seem to be critical for the proper progress of pregnancy, and are therefore favorable targets. We suppose that knockdown of selected genes may be a major improvement over existing SIT, which suffers from the reduced fitness of sterilized males caused by irradiation [53]. Given the higher FCs in uterus, targeting gene expression in this tissue may result in a more evident reduction of fecundity. However, ovaries should not be neglected, as manifested by several genes in Table 5 involved in early developmental stages. Apart from focusing on the highest fold changes, neuropeptide receptors with moderate increases in expression should be also verified as targets, given their roles in mediating the tissue control.

## Additional files

Additional file 1: **Table S1.** *G. m. morsitans* genes and homologues. *G. m. morsitans* gene IDs, gene names, and gene descriptions, and homologues from *D. melanogaster*. (XLSX 788 kb)

Additional file 2: **Table S2.** Analysis - ovaries. Counts of ovarian reads uniquely mapped to annotated genes, and calculated *P*-values and fold changes. Includes lists of fold changes arranged in descending order. (XLSX 1150 kb)

Additional file 3: **Table S3.** Analysis - uteri. Counts of uterine reads uniquely mapped to annotated genes, and calculated *P*-values and fold changes. Includes lists of fold changes arranged in descending order. (XLSX 1130 kb)

Additional file 4: **Table S4.** *P*-values and fold changes of neuropeptide receptor genes. Ovarian and uterine *P*-values and fold changes of 33 putative neuropeptide receptor genes. (XLSX 1310 kb)

## Abbreviations

AAT: animal African trypanosomiasis
FC: fold change
HAT: human African trypanosomiasis
MGP: Milk Gland Protein
SIT: Sterile insect technique
